# Exploring the Interobserver Agreement in Computer-Aided Radiologic Tumor Measurement and Evaluation of Tumor Response

**DOI:** 10.3389/fonc.2021.691638

**Published:** 2022-01-31

**Authors:** Hongsen Li, Jiaying Shen, Jiawei Shou, Weidong Han, Liu Gong, Yiming Xu, Peng Chen, Kaixin Wang, Shuangfeng Zhang, Chao Sun, Jie Zhang, Zhongfeng Niu, Hongming Pan, Wenli Cai, Yong Fang

**Affiliations:** ^1^ Department of Medical Oncology, Sir Run Run Shaw Hospital, Zhejiang University School of Medicine, Hangzhou, China; ^2^ Quantilogic Healthcare Zhejiang Co. Ltd, Hangzhou, China; ^3^ School of Medical Imaging, Shanghai University of Medicine and Health Sciences, Shanghai, China; ^4^ Department of Radiology, Sir Run Run Shaw Hospital, Zhejiang University School of Medicine, Hangzhou, China; ^5^ Department of Radiology, Massachusetts General Hospital, Harvard Medical School, Boston, MA, United States

**Keywords:** tumor measurements, evaluation agreement, response evaluation criteria in solid tumors (RECIST), measurement variability, treatment assessment

## Abstract

The accurate, objective, and reproducible evaluation of tumor response to therapy is indispensable in clinical trials. This study aimed at investigating the reliability and reproducibility of a computer-aided contouring (CAC) tool in tumor measurements and its impact on evaluation of tumor response in terms of RECIST 1.1 criteria. A total of 200 cancer patients were retrospectively collected in this study, which were randomly divided into two sets of 100 patients for experiential learning and testing. A total of 744 target lesions were identified by a senior radiologist in distinctive body parts, of which 278 lesions were in data set 1 (learning set) and 466 lesions were in data set 2 (testing set). Five image analysts were respectively instructed to measure lesion diameter using manual and CAC tools in data set 1 and subsequently tested in data set 2. The interobserver variability of tumor measurements was validated by using the coefficient of variance (CV), the Pearson correlation coefficient (PCC), and the interobserver correlation coefficient (ICC). We verified that the mean CV of manual measurement remained constant between the learning and testing data sets (0.33 vs. 0.32, *p* = 0.490), whereas it decreased for the CAC measurements after learning (0.24 vs. 0.19, *p* < 0.001). The interobserver measurements with good agreement (CV < 0.20) were 29.9% (manual) vs. 49.0% (CAC) in the learning set (*p* < 0.001) and 30.9% (manual) vs. 64.4% (CAC) in the testing set (*p* < 0.001). The mean PCCs were 0.56 ± 0.11 mm (manual) vs. 0.69 ± 0.10 mm (CAC) in the learning set (*p* = 0.013) and 0.73 ± 0.07 mm (manual) vs. 0.84 ± 0.03 mm (CAC) in the testing set (*p* < 0.001). ICCs were 0.633 (manual) vs. 0.698 (CAC) in the learning set (*p* < 0.001) and 0.716 (manual) vs. 0.824 (CAC) in the testing set (*p* < 0.001). The Fleiss’ kappa analysis revealed that the overall agreement was 58.7% (manual) vs. 58.9% (CAC) in the learning set and 62.9% (manual) vs. 74.5% (CAC) in the testing set. The 80% agreement of tumor response evaluation was 55.0% (manual) vs. 66.0% in the learning set and 60.6% (manual) vs. 79.7% (CAC) in the testing set. In conclusion, CAC can reduce the interobserver variability of radiological tumor measurements and thus improve the agreement of imaging evaluation of tumor response.

## Introduction

Radiological imaging examination plays an important role in monitoring of tumor progression or evaluation of tumor response to treatment in oncological clinical trials and clinical care (1–4). Cancer patients may undergo longitudinally radiological imaging examinations, such as CT (computed tomography), MRI (magnetic resonance imaging), and/or PET (positron emission tomography), to quantify tumor burden for assessment of tumor response to treatment (4). The criteria for evaluation of tumor response vary in terms of tumor types and treatment methods. In 1981, the World Health Organization (WHO) published the first criteria for solid tumor response evaluation, which adopted bidimensional measurement as the tumor imaging biomarkers for quantifying tumor burden (5). The Response Evaluation Criteria in Solid Tumors (RECIST) published in 2000 and its revised version (RECIST 1.1) in 2009 adopted the unidimensional instead of bidimensional measurement as the tumor imaging biomarkers to quantifying tumor burden (6). Nowadays, oncology clinical trials increasingly rely on image-based surrogate endpoints. RECIST represents the internationally recognized evaluation criteria for solid tumors (5, 6). With the advent of oncologic therapies (targeted therapy, immunotherapy, etc.), the radiological response assessment criteria are also evolving, for instance, the modified RECIST (mRECIST) for evaluating the response of primary hepatocellular carcinoma (HCC) to targeted therapy (5, 7) and the modified RECIST for assessment of cancer immunotherapy (iRECIST) (8). Moreover, the FDA published guidelines in 1994, with updates in 2004 (9) and 2019 (10) for standardizing the radiological assessment of tumor responses as a primary endpoint in clinical trials.

Reliable evaluation of tumor response depends on two aspects: the correct selection of target lesions and the accurate and reproducible measurement of tumor burden. Although the concept concerning the measurement of the maximal tumor diameter is undoubtedly simple and convenient for physicians, has a long history of clinical applications, and is familiar to management agencies (FDA), the methodology of measuring lesions is poorly defined in RECIST or WHO. The subjective linear measurement has been criticized for its low reproducibility and high inter- and intra-observer variabilities of the tumor response assessment. Several studies observed that the intraobserver variability was among 6% to 14%, and the interobserver variability was approximately 10% to 25% (11). These measurement variabilities may lead to a misinterpretation of tumor response, in particular the large interobserver variability. Some studies found that the misclassification of tumor responses caused by the interobserver measurement variabilities was as high as 43% (WHO) and 30% (RECIST) (12). A meta-analysis summarized the RECIST-based observer variability of manual measurements on CT images (13): relative measurement differences ranged from 17.8% to 16.1% for the same observers (5 studies, 648 lesions measured), −22.1% to 25.4% between two observers (8 studies, 1,878 lesions measured), and −31.3% to 30.3% among multiple observers (3 studies, 575 lesions measured). It has been reported that even for expert radiologists, there was considerable variability in interpretation of lesion boundaries, in particular for irregular lesions, with the interobserver variability accounting for 40% of a lesion size (14), which may inevitably result in significant difference in tumor size measurements (12). Because this interobserver variability in tumor measurements may lead to a misclassification of tumor growth rate or response even for the same selected target lesions, the methodology for measuring lesions needs to be improved (15).

The aims of this study therefore were (1) to develop a computer-aided contouring (CAC) tool for interactive measurement of maximum tumor diameter required by RECIST or WHO criteria, (2) to validate the interobserver agreement of the CAC tool in tumor diameter measurements, and (3) to assess the consistency of the CAC tool in the evaluation of tumor response in terms of RECIST 1.1 criteria.

## Materials and Methods

**Figure 1:**
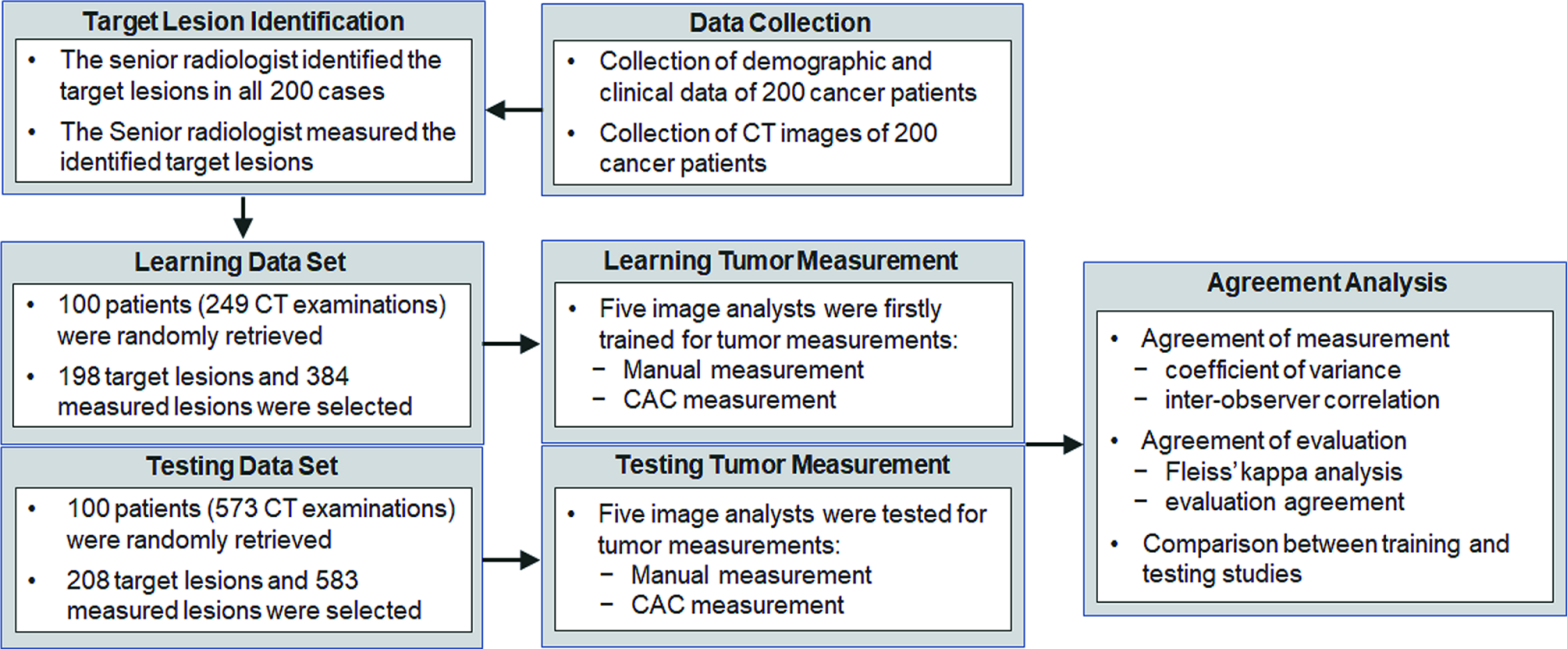
The flow chart of the study. Data collection and the design of the study.

The institutional ethics committee has approved this retrospective study, in which informed consent was waived, but patient confidentiality was protected. The study design is illustrated in **Figure 1**.

### Patient Cohort

This study was approved by the ethics committee of the Sir Run Run Shaw Hospital, Zhejiang University School of Medicine (Scientific Research No.2020617-33). The clinical information used in this study was previously archived data, files, records, pathological specimens, and diagnostic specimens, and was approved of exempting informed consent by Institutional Review Board (IRB). Two hundred patients with pathological confirmed cancers between January 1, 2015 and December 31, 2019 enrolled in several Phase I or II oncology clinical trials evaluating systemic therapies in lung, liver, and colorectal cancer patients were retrospectively selected in a random sample from the hospital medical records system. Inclusion criteria for this study were as follows: (1) an adult patient over 18 years old; (2) pathological confirmed malignant solid tumors in head and neck, lung, liver, abdomen, lymph nodes, and other body parts; (3) received anti-cancer treatment such as radiotherapy, chemotherapy, targeted therapy, etc.; (4) performed contrast-enhanced CT examinations before and after treatment; and (5) had at least one measurable lesion ≥10 mm in terms of RECIST 1.1 criteria.

The exclusion criteria were as follows: (1) patients had no measurable lesions defined by RECIST criteria; (2) patients had different image modalities other than CT before or after treatment; (3) the imaging examination did not cover the entire lesion; and (4) incomplete clinical records, for instance, the date of treatment and missing CT images.

### Imaging Examination

Contrast-enhanced CT examinations were acquired on multi-detector CT scanners (GE, Siemens) with a tube voltage of 120 kVp, an automatic tube current, a slice collimation of 0.6 to 1.5 mm, and standard reconstruction kernel and slice thickness ranging from 1.25 to 5 mm defined in the clinical trial protocols.

### Lesion Selection and Lesion Size Measurement

According to the requirements of target lesion selection in RECIST 1.1 (6), a senior radiologist selected up to 5 measurable lesions per patient with a maximum of 2 lesions per organ as the target lesions. Although RECIST 1.1 defines that the target lesions at baseline should be ≥10 mm in the longest diameter for extranodal disease and ≥15 mm in the short-axis diameter for nodal disease, some target lesions may be smaller than 10 mm on follow-up examinations due to treatment effects. For the purpose of treatment response evaluation, those <10 mm lesions in follow-up examinations were also included in the study to assess the variability. Each selected lesion was independently measured using a manual tool and a CAC tool by five image analysts, respectively. Furthermore, to reduce the variability caused by subjective selection of target lesions, we recruited a senior radiologist (ZN) to identify these target lesions using an arrow to enable the recognition by the image analysts. The image analysts were informed that the arrow was randomly marked on one of the slices of the lesion and thus did not indicate the slice with the longest lesion diameter. The image analysts were required to determine the slice for longest lesion diameter based on their own judgements after examining all the slices of the lesion.

In terms of the RECIST 1.1, the longest diameter of a target lesion was measured on the transverse (axial) plane in CT by manual method first. Each image analyst examined each target lesion selected by the senior radiologist and determined the transverse (axial) plane with the longest diameter based on visual assessment. Standard window/level (HU) settings were applied in terms of organs or body parts, for instance, lung (1500/−500), liver (310/80), abdomen (400/60), and neck (250/30). The longest diameter of each lesion was measured by placing two endpoints of the diameter on the edge of the lesion without crossing normal tissues.

Following the manual measurement, each image analyst was required to measure the lesion by using the CAC tool, which detected the optimal boundaries in terms of the initial contours given by the image analyst. An image analyst reviewed the CAC-generated contour and corrected it if it was unsatisfactory. The longest diameter (for extranodal disease) or the longest perpendicular diameter (nodal disease) of the lesion was automatically estimated by the CAC tool in either 2D/3D mode: if only one slice of a lesion was contoured, the longest diameter of the contour was directly calculated; if multiple slices of a lesion were contoured, the contour with the maximum cut-plane area was first selected and the longest diameter of that contour was calculated. This diameter served as the CAC measurement of a lesion.

### Computer-Aided Contouring Method

**Figure 2:**
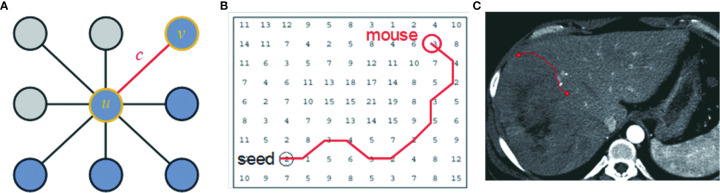
The local boundary is defined as the optimal path with the minimum cost between two corresponding nodes in the graph. **(A)** An image can be represented as a 2D weighted bi-directed graph, in which one node corresponds to a pixel in the image. Each node (pixel) has 8 links (edge) connecting to its neighborhood pixels. Both node (*u* or *v*) and link **(C)** have a related cost. **(B)** A contour on a 2D transversal image is defined as the optimal path with the minimum cost between two corresponding nodes in the graph, which can be searched by dynamic-programming methods such as Dijkstra’s algorithm. **(C)** A contour in the image corresponds to the optimal path in **(B)**.

CAC is a computer-aided contouring toolkit that we developed based on the optimal path search using the dynamic-programming techniques in graph theory (15). A transversal image can be represented by a 2D weighted bi-directed graph, in which one node corresponds to a pixel in the image. Each node (pixel) has 8 connecting links (edges) to its neighboring nodes (pixels), and each node and link has an associated cost. The local boundary of a region-of-interest (ROI) on a 2D transversal image is defined as the optimal path with the minimum cost between two corresponding nodes in the graph, as illustrated in **Figure 2**.

Dynamic-programming theory indicates that the optimal path between node *u* and *v* is either a direct link between *u* and *v*, or going from *u* to some node *w* and then directly from node *w* to *v*, which is also named Dijkstra’s observation (16). Based on this theory, Dijkstra’s algorithm first computes the minimum cost path from the seed node *u* to every node in the entire graph. This set of minimum paths can be represented as a tree structure. Once this tree is established, the optimal path from any node to the seed node *u* can be extracted in the tree in real time by traversing from root *u* to any node in the tree. For a 512 × 512 image, it takes less than 1 s to establish the tree structure from the seed node to the entire image. As long as the tree structure is established, the traversing from the root to a node in the tree structure is very efficient, less than 1 ms. This ensures the real-time interactivity of the CAC algorithm.

**Figure 3:**
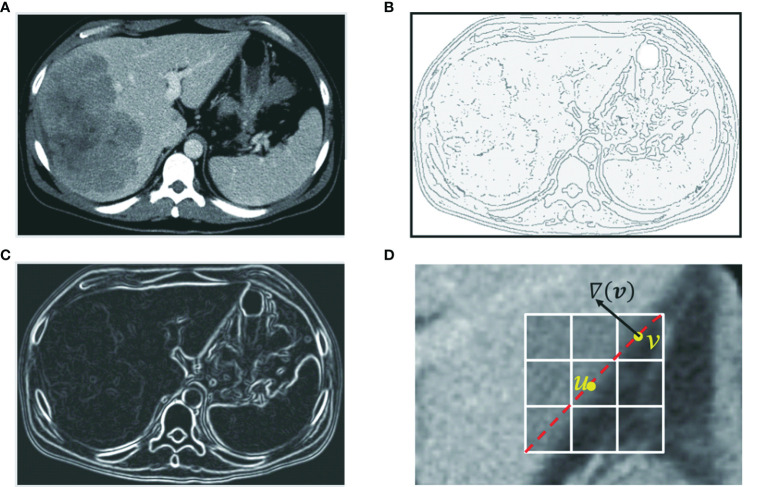
Edge features such as zero-crossing and gradient are incorporated into the computation of local cost. **(A)** Original axial image. **(B)** Zero-crossing: points with local maximal gradient magnitude corresponds to a local boundary point with lower cost. **(C)** Gradient magnitude: a point with a large gradient magnitude tends to indicate a local boundary and thus a lower cost. **(D)** Gradient direction: a local boundary is perpendicular to the gradient direction. Cost is low if the direction of a link (uv) is perpendicular to the gradient direction [▽(*v*)] of the pixel.

Because an optimal path corresponds to a segment of ROI boundaries, pixels or links between neighboring pixels that exhibit strong edge features are made to have low local costs. Edge features such as Laplacian zero-crossing *S_ZX_* (*v*), gradient magnitude *S*_g_(*v*), and gradient direction *S_d_* (*u*,*v*) (17) are incorporated into the computation of local cost. The cost going from node (pixel) *u* to node (pixel) *v* is a weighted sum of each corresponding local cost defined as *c*(*u*, *v*) = *ω*_ZX_ · *S_ZX_* (*v*) + *ω*_g_ · *S_g_* (*v*) + *ω*_d_ · *S_d_* (*u*,*v*), where *ω*_ZX_, *ω*_g_, and *ω*_d_, are constants to weight features. In **Figure 3**, we demonstrate these cost functions.

**Figure 4:**
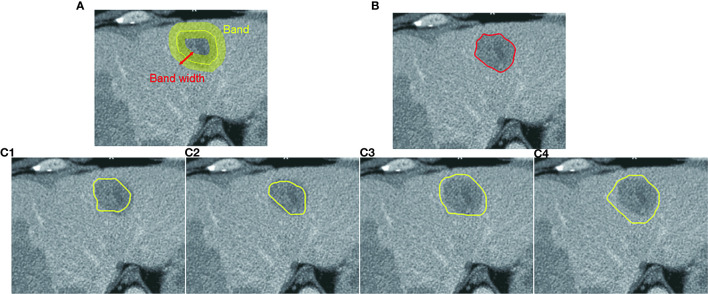
Computer-aided contouring (CAC) tool. **(A)** Tumor boundary is searched within a band centered by the mouse moving trajectory. **(B)** The resulting boundary is the optimal path searched in terms of the minimum cost of local edges. **(C1–C4)** Different mouse moving trajectories result in the same tumor boundary shown in **(B)** after applying the CAC tool.

In CAC, we assume that the boundary of a ROI is located within the neighborhood of the mouse moving trajectory. This neighborhood is defined as a band on the transversal images centered by the mouse moving trajectory, as shown in **Figure 4A**. The width of the band can be adjusted in terms of the size of the segmented ROIs. In our study, we set the band width to be 20 pixels or 10 pixels on both sides of the initial mouse moving trajectory. The local boundary of a ROI will be searched within this local band. The use of a local band significantly reduces the searching space of optimal path. More importantly, it may improve the stability of CAC detected boundary by converging to the optimal tumor boundary and ignoring nodes or links outside the band, as demonstrated in **Figures 4C1**–**C4**.

To reduce the influence of inaccurate positions of initial seed points (such as *u*, *v* given by a user) and ensure that the selected seeds are at or near the boundary of the ROI, we applied a seed selection process *via* a two-pass optimal path searching scheme: after the first optimal path searching using initial given seeds, the points with the lowest cost in every segments of the optimal path between two initial seed points are selected as the new seed points for the second searching of the optimal path. This seed selection process significantly reduces the inaccuracy and the interobserver variability of seed positioning. Because the path is piece-wise optimal (i.e., optimal between seed points), the detected boundary using selected seed points is stable and reproducible, as demonstrated in **Figure 4** wherein the different trajectories in **Figures 4C1**–**C4** result in the exact same contour shown in **Figure 4B**.

**Figure 5:**
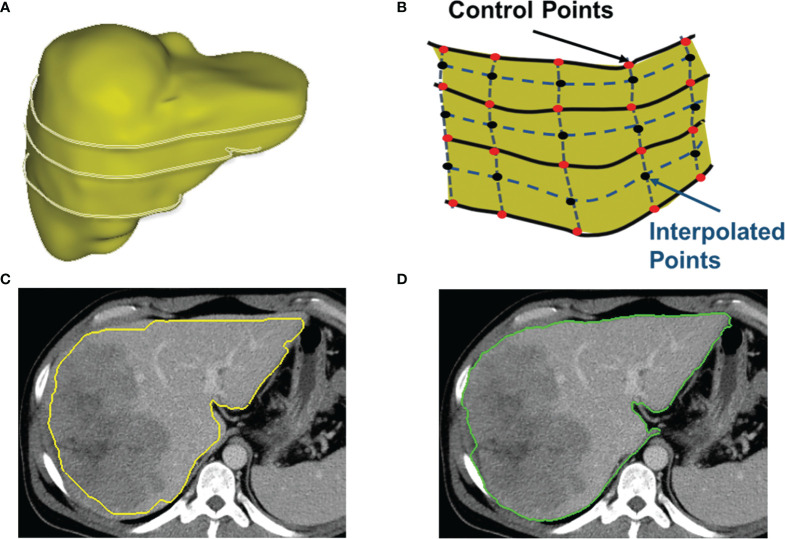
Detection of between-slices contours by interpolation and optimization. **(A)** A 3D shape of a liver with contours on several non-adjacent key slices. CAC toolkit can detect the contours between key slices. **(B)** The interpolation of control points on slices between the key slices. **(C)** The initial contour estimated by using interpolated control points. **(D)** The resulting contour generated by the two-pass optimal path searching scheme using the estimated contour in **(C)**.

CAC provides the ability to detect (searching and snapping) the ROI boundaries between key slices that have user-defined ROI contours. In general, the user contoured a lesion at the first and the last slice, and for the middle key slices of a lesion, the user may contour at every 3–5 slice intervals along the scanning direction. The exact number of slices between key slices depended on the complexity (irregularity, infiltration, and inhomogeneity) of the lesion. The contours between these key slices were detected in an automated manner by using the scheme of between-slice contour detection in CAC, as illustrated in **Figures 5A, B**. This automated between-slice detection scheme consists of three steps below:

(1) Interpolate seed points on each of the between-slices using a pair of neighboring key slices;
(2) Estimate initial contours by connecting these interpolated seed points; and
(3) Search the local boundary using the two-pass optimal path searching scheme (described above) within the band of the estimated contour.

**Figure 5C** shows an initial estimated contour created by interpolation step, and **Figure 5D** is the resulting contour generated by the two-pass optimal path searching scheme in the band of the initial contour in **Figure 5C**. Because our two-pass optimal path searching scheme can find accurate and stable ROI boundaries in an automated manner, the between-slices contour detection can significantly reduce the user time and effort in interactive contouring while still achieving an accurate segmentation. This volumetric contouring ensures that CAC can estimate the longest diameter of the contour with the maximum cut-plane area of a lesion, which is called the 3D mode for longest diameter estimation.

Based on this theory, we developed a CAC toolkit for interactive segmentation of tumors and organs, which includes (1) real-time searching of the local boundary on a 2D transversal image, (2) automated detection of between-slice contours for volumetric contouring, and (3) automated calculation of the longest diameter (RECIST criteria) or the product of perpendicular longest diameters (WHO criteria) of a tumor on either one transversal image (2D mode) or multiple transversal images (3D mode).

### Validation Scheme

These 200 patients were randomly divided into two data sets of 100 patients for experiential learning set (data set 1) and testing (data set 2) of tumor measurement using manual and CAC tools. Five image analysts who had no prior knowledge of tumor measurements and RECIST criteria were first instructed how to measure a lesion diameter in terms of the RECIST 1.1 criteria in the learning set, and then tested in the second data set by using manual and CAC tools on the V3D platform (Quantilogic Healthcare). Tumor responses were evaluated in terms of RECSIT 1.1 criteria by using the resulting measurements of the manual and CAC tools, respectively. The reliability and reproducibility of tumor measurement and response evaluation were compared between manual and CAC tools.

### Data Analysis

To evaluate the agreement of the measurement, we calculated the coefficient of variance (CV) of five measurements of each tumor, the correlation between any two pairs of analysts [Pearson correlation coefficient (PCC)], and the interobserver correlation coefficient [interclass correlation coefficient (ICC)] of five image analysts. To evaluate the agreement of tumor response evaluation, we calculated the Fleiss’ kappa coefficients.

Statistical analysis was conducted by R software, version 3.3.2 (https://www.R-project.org/), and SPSS Statistics for Windows, version 17. 0 (SPSS Inc., Chicago, Ill., USA), of which correlations were calculated by SPSS and other statistics were calculated by R. Quantitative variables were shown as mean ± SD. Statistical group comparisons of data were analyzed by Wilcoxon rank-sum (continuous variables) test and *χ* ^2^ (categorical/dichotomous variables) test. A Student’s *t*-test was used for the continuous variable. A *p*-value less than 0.05 was considered statistically significant.

## Results

### Clinical Data

Two hundred patients were randomly separated into two sets of 100 patients: data set 1 (71 males; 29 females; mean age 58.7 years; range 34 to 73 years) and data set 2 (67 males; 33 females; mean age 57.1 years; range 21 to 74 years). A total of 249 CT examinations were retrieved from PACS in the first data set, and 573 CT examinations were retrieved in the second data set.

**Figure 6:**
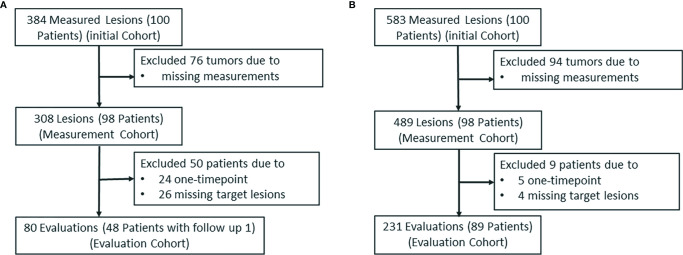
The patient selection and distribution. The patient selection and distribution in **(A)** the learning study and **(B)** the testing study.

A total of 384 and 583 reported tumors were identified by a senior radiologist (ZN) in data sets 1 and 2, respectively. Because of one or more missing measurements in the resulting measurements by the five image analysts, 76 and 94 tumors were excluded in data sets 1 and 2, respectively. This resulted in 308 lesions in data set 1 and 489 lesions in data set 2 in the measurement study. In the tumor response evaluation study, we excluded patients caused by one time point and missing target lesions in the follow-up examinations. This resulted in 80 evaluations (48 patients) and 231 evaluations (89 patients) in data sets 1 and 2, respectively. **Figure 6** shows the patient selection and distribution in data sets 1 and 2 for the learning and testing studies.

Overall, the lesion diameters ranged from 10.0 to 105.0 mm (mean: 30.7 ± 19.0 mm, median 25.5 mm) in data set 1 and 5.0 to 88.0 mm (mean: 22.5 ± 15.1 mm, median 18.0 mm) in data set 2. In the tumor response evaluation study, the average number of follow-up examinations was 1.6 and 2.6 in the learning and testing study, and the average number of lesions per patient was 1.94 and 1.92 in two sets, respectively.

**Figure 7:**
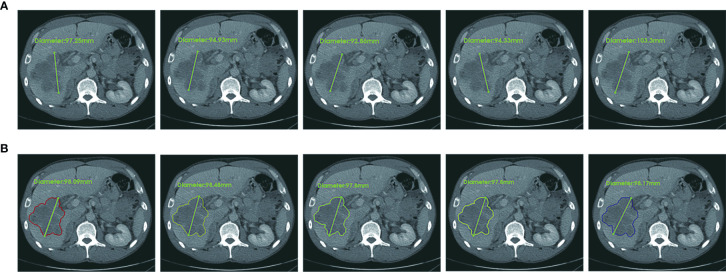
An example case of manual and CAC measurements. In terms of RECIST 1.1 criteria, the longest diameter of a target lesion is measured on the image acquisition plane (axial plane in CT). **(A)** Upper row: five manual measurements showed the mean value 96.5 ± 4.1 mm, range 92.7 mm to 103.3 mm, CV (coefficient of variance) 4.3%. **(B)** Lower row: five CAC measurements showed the mean value 98.1 ± 0.3 mm, range 97.8 mm to 98.1 mm, CV 0.3%. The agreement of CAC measurements was substantially better than that of manual measurements.

The details of the patient characteristics and lesion statistics are summarized in **Table 1**. The example case displayed in **Figure 7** demonstrates a significant reduction of interobserver variability by the CAC tool.

**Table 1:** The demographic and clinical characteristics of the experiential learning data set (data set 1) and testing data set (data set 2).

Group	Data set 1 (*n* = 100)	Data set 2 (*n* = 100)	*p-*value
Sex			
Female	29	33	0.647
Male	71	67	
Enrolled age			
Mean	58.7	57.1	0.268
Range (Min–Max)	34–73	21–74	
Treatment			
Chemotherapy only	8	12	0.505
TKIs based regimes	33	36	
Antibodies based regimes	59	52	
Target lesions			
Total	198	208	
Lung	66	78	0.052
Lymph nodes	60	58	
Liver	38	20	
Body wall	10	16	
Soft tissue	7	7	
Other	17	29	
Adrenal gland	1	11	/
Peritoneum	5	5	
Brain	3	2	
Breast	3	2	
Kidney	1	2	
Annex	0	2	
Pancreas	2	0	
Spleen	1	1	
Bone	0	1	
Diaphragm	0	1	
Limbs	0	1	
Parotid gland	0	1	
Pharynx	1	0	

### Agreement of Measurement and Analysis of Coefficient of Variance

**Figure 8:**
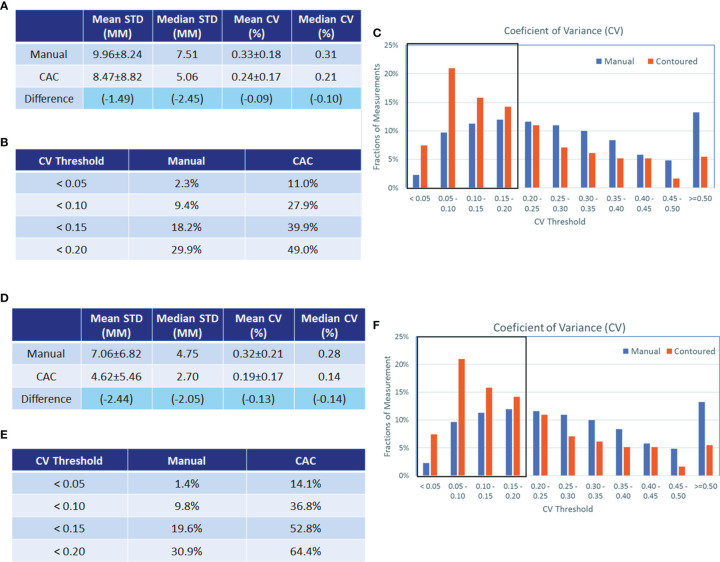
Analysis of coefficients of variance (CV) in the experiential learning and testing study. Analysis of coefficients of variance (CV) in the experiential learning study (data set 1, **A–C**) and in the testing set (data set 2 **D–F**). The standard deviation (STD) and CV of the manual and CAC measurements (mean and median values given in a and d, respectively) and the CV distribution (**B**, **E**, respectively) are shown before and after learning. The boxplots in **(C, F)** show the fractions of measurements with good agreement (<0.2). In data set 1, this was 29.9% vs. 49.0% for manual and CAC measurement, respectively, and in data set 2, this was 30.9% vs. 64.4% for manual and CAC measurements, respectively.

**Figures 8A–C** show that the CV in the learning set was reduced by CAC compared to manual measurements, and the percentage of measurements with a CV of <0.2 were 29.9% (manual) vs. 49.0% (CAC), which was statistically significant (*p* < 0.001). **Figures 8D–F** show the CV in the testing set after experiential learning. It indicates that while the mean CV of manual measurement remained constant between the first and second data sets (0.33 vs. 0.32, *p* = 0.490), it decreased for the CAC measurements after learning (0.24 vs. 0.19, *p* < 0.001) as the image analysts became familiar with the CAC tool. In addition, we measured lesions from different body parts in the study to demonstrate the generalizability of the CAC tool. Overall, the CAC tool outperformed manual measurement at all six body parts: CV between manual and CAC tools were 0.27 vs. 0.20 (*p* = 0.004) (lung), 0.23 vs. 0.17 (*p* = 0.046) (liver), 0.30 vs. 0.25 (*p* = 0.240), (abdomen), 0.30 vs. 0.20 (*p* < 0.001) (lymph), and 0.37 vs. 0.27 (*p* = 0.003) (other), respectively. This demonstrated that the CAC tool is a general-purpose imaging tool for tumor measurements, indicating the great potentials for the clinical adoption of the CAC tool.

### Analysis of Interobserver Correlation

**Table 2** presents the PC matrix among five image analysts in data sets 1 and 2, respectively. After experiential learning, we observed that the agreement of each analyst with four other image analysts was improved. However, the CAC tool still outperformed the manual tool. In addition, the two-way mixed interobserver correlation analysis revealed the ICC values of 0.633 (manual) vs. 0.698 (CAC) in data set 1, and 0.716 (manual) vs. 0.824 (CAC) in data set 2. After experiential learning, the CAC tool achieved “excellent” level of agreement (ICC > 0.75).

**Table 2:** Pearson correlation matrix among five image analysts in data sets 1 and 2.

Data Set 1 (Learning)										
	(A) Manual measurement					(B) CAC measurement				
	Analyst 1	Analyst 2	Analyst 3	Analyst 4	Analyst 5	Analyst 1	Analyst 2	Analyst 3	Analyst 4	Analyst 5
Analyst 1	1	0.385	0.418	0.367	0.401	1	0.538	0.533	0.507	0.565
Analyst 2	0.385	1	0.418	0.367	0.401	0.538	1	0.82	0.702	0.895
Analyst 3	0.418	0.808	1	0.736	0.865	0.533	0.82	1	0.728	0.909
Analyst 4	0.367	0.667	0.736	1	0.673	0.506	0.702	0.728	1	0.752
Analyst 5	0.401	0.871	0.865	0.673	1	0.565	0.895	0.909	0.752	1
Correlation with Others	0.39	0.68	0.61	0.54	0.59	0.54	0.74	0.75	0.67	0.78
Data Set 2 (Testing)										
	(C) Manual measurement					(D) CAC measurement				
	Analyst 1	Analyst 2	Analyst 3	Analyst 4	Analyst 5	Analyst 1	Analyst 2	Analyst 3	Analyst 4	Analyst 5
Analyst 1	1	0.546	0.672	0.605	0.639	1	0.760	0.815	0.815	0.845
Analyst 2	0.546	1	0.823	0.741	0.796	0.760	1	0.850	0.795	0.828
Analyst 3	0.672	0.823	1	0.796	0.846	0.815	0.850	1	0.830	0.883
Analyst 4	0.605	0.741	0.796	1	0.814	0.815	0.795	0.830	1	0.931
Analyst 5	0.639	0.796	0.846	0.814	1	0.845	0.828	0.883	0.931	1
Correlation with Others	0.62	0.73	0.78	0.74	0.77	0.81	0.81	0.84	0.84	0.87

**(A)** The manual measurement: The mean Pearson correlation coefficient (PCC) was 0.56 ± 0.11, range 0.39 to 0.69. **(B)** The CAC measurement: The mean PCC was 0.69 ± 0.10, range 0.54 to 0.78 in data set 1. The two-way mixed interobserver correlation coefficient (ICC) was 0.633 (manual) vs. 0.698 (CAC). **(C)** The manual measurement: The mean Pearson correlation coefficient (PCC) was 0.73 ± 0.07, range 0.62 to 0.78. **(D)** The CAC measurement: The mean PCC was 0.84 ± 0.03, range 0.81 to 0.87 in data set 2. The two-way mixed interobserver correlation coefficient (ICC) was 0.716 (manual) vs. 0.824 (CAC). CAC tool was in “excellent” agreement (>0.75), whereas manual method remains in “good” agreement.

### Agreement of Tumor Response Evaluation

Tumor response was evaluated in four categories: complete response (CR), partial response (PR), stable disease (SD), and progressive disease (PD). As shown in **Table 3**, the total numbers of RECIST evaluations were 80 and 231 in the two data sets, respectively. The Fleiss’ kappa analysis revealed that the percentage overall agreement (*K*) was 58.7% (manual) vs. 58.9% (CAC) in the first learning set, whereas it was 62.9% (manual) vs. 74.5% (CAC) in the second testing set. The agreement increased approximately 4.2% in manual and 15.6% in CAC. The CAC tool approached “excellent” agreement (*K* > 0.75), whereas the manual method remained in “good” agreement.

**Table 3:** Agreement of RECIST 1.1 evaluation.

	(A) Data set 1 (Learning)				(B) Data set 2 (Testing)			
	Total #	Votes%	Cases #	Cases%	Total #	Votes%	Cases #	Cases%
Manual	80	80%	44	55.00%	231	80%	140	60.60%
		100%	22	27.50%		100%	77	33.30%
CAC	80	80%	52	66.00%	231	80%	184	79.70%
		100%	17	21.30%		100%	113	48.90%

**(A)** Agreement in data set 1. **(B)** Agreement in data set 2.

Among five tumor response evaluations by five image analysts for each patient, we calculated the “excellent” patient-level agreement, which was defined as the same response category as assessed by more than four image analysts assessed the same response categories, i.e., more than 80% evaluation results were the same. The manual measurements achieved a constant level of agreement: 55.0% (learning) and 60.6% (testing) (*p* < 0.001), whereas CAC measurements improved from 66.0% (learning) to 79.7% (testing) (*p* < 0.001). It indicates that when image analysts became familiar with the CAC tool after experiential learning, the difference of agreement between two tools on tumor response evaluation becomes more significant, increasing from 11.0% [learning: 66.0% (CAC) vs. 55.0% (manual), *p* < 0.001] to 19.1% [testing: 79.7% (CAC) vs. 60.6% (manual), *p* < 0.001].

## Discussion

This study shows the improvements in unidimensional tumor measurements that can be gained by utilizing a CAC tool compared to manual methods and its impact on response evaluation. In order to prevent bias from prior knowledge or practice, we recruited five image analysts who were unfamiliar with tumor measurement and response assessment, and were instructed to learn and subsequently tested independently the measurement of longest lesion diameter in terms of RECIST criteria using manual and CAC tools. For this purpose, we divided the 200 patients into two sets of 100 patents for learning and testing.

This study demonstrated that the interobserver agreement of the manual measurement had approximately 1/3 of the cases, whereas the CAC tool achieved twice the better performance, which was also indicated by the “excellent” level of ICC of the CAC tool. The novelty of the CAC method is its reproducibility and consistency of segmentation, which can significantly reduce the interobserver variability in tumor measurement, thereby ensuring the quality and repeatability of tumor response evaluation among different radiologists and institutions. Manual contouring tools are easy to use but very time-consuming, whereas semi-automated interactive tools (such as snake and speedline) are less labor-intensive but highly unstable. We thus developed the CAC tool in the context of tumor response assessment for the purpose of an efficient and consistent tumor measurement. Compared with other open-source segmentation tools in ITK Snap (www.itksnap.org) and Seg3D (www.sci.utah.edu/cibc-software/seg3d.html), the CAC tool has certain advantages on accuracy and reproducibility. A comparison of image contouring tools with the CAC tool is given in **Table S1**.

This study had several limitations. The first limitation was the single CT modality that we used in the validation study. We selected CT examinations for the experiential learning and testing in order to demonstrate the feasibility of the CAC tool. However, the CAC tool is not limited to CT modality. We will investigate the reliability and reproducibility of the CAC tool in MRI and PET/CT examinations. Another limitation is that our study used single-center data from one institute. We plan to collect multi-center cases to validate the reproducibility of the CAC tool for tumor measurements and evaluation. The CAC tool needs multi-center and prospective data for further validation and improvements. Although the clinical significance of the CAC tool warrant validation by larger multi-center studies, it may provide a reliable and reproducible solution for radiological tumor measurement, which may further affect the imaging endpoints in tumor response evaluation.

In conclusion, our study demonstrated that the computer-aided contouring method can significantly improve the agreement of radiologic tumor measurements, reduce the interobserver variability of tumor measurement, and thus improve the agreement of tumor response evaluation in oncology clinical trials and clinical care.

## Data Availability Statement

The raw data supporting the conclusions of this article will be made available by the authors, without undue reservation.

## Ethics Statement

The studies involving human participants were reviewed and approved by the Ethics committee of the Sir Run Run Shaw Hospital, Zhejiang University School of Medicine. Written informed consent for participation was not required for this study in accordance with the national legislation and the institutional requirements. Written informed consent was not obtained from the individual(s) for the publication of any potentially identifiable images or data included in this article.

## Author Contributions

Conceptualization: HP, WC, YF, and HL. Investigation and data collection: HL, JWS, WH, and LG. Analysis: JYS, YX, and ZN. Image analysist: PC, KW, SZ, CS, and JZ. Wrote original draft: JYS and WC. Review, editing, and final approval: WC, YF, and HP. Supervision: YF. All authors contributed to the article and approved the submitted version.

## Funding

This study was partly supported by Natural Science Foundation of Zhejiang Province [Grant Numbers LY13H160013 and LQ16H160003], Health Commission of Zhejiang Province [Grant Number 2016KYA115], Medical Science and Technology Project of Zhejiang Province [Grant Numbers 2016ZDB007, 2017197380, and 2017ZD021], CSCO Health Project [Y-QL2019-0316 and Y-MSD2020-0314], the Zhejiang Medical Innovative Discipline Construction Project-2016, and China International Medical Fund Exchange Conference [XS022].

## Conflict of Interest

Author YX was employed by the company Quantilogic Healthcare Zhejiang Co. Ltd.

The remaining authors declare that the research was conducted in the absence of any commercial or financial relationships that could be construed as a potential conflict of interest.

## Publisher’s Note

All claims expressed in this article are solely those of the authors and do not necessarily represent those of their affiliated organizations, or those of the publisher, the editors and the reviewers. Any product that may be evaluated in this article, or claim that may be made by its manufacturer, is not guaranteed or endorsed by the publisher.
