# Supplementary material for: Exploring the Interobserver Agreement in Computer-Aided Radiologic Tumor Measurement and Evaluation of Tumor Response
Source: Front Oncol. 2022 Jan 31;11:691638. doi: 10.3389/fonc.2021.691638 (PMC8841678; doi:10.3389/fonc.2021.691638)
Supplement: Supplementary file 1 [file Table_1.doc]

**Table** **S1.** Evaluation of the performance of the CAC toolkit by comparison of our CAC method with five liver segmentation methods selected from ITK-Snap (www.itksnap.org) and Seg3D (www.sci.utah.edu/cibc-software/seg3d.html) in terms of accuracy score, running time, and ease of use by using 10 liver cases downloaded from MICCAI database (www.sliver07.org).

| Method (Software) | Average Score | SD of Score | Running time (minutes) | SD of Time (minutes) | Easy-of-Use |
| --- | --- | --- | --- | --- | --- |
| Manual Contouring (ITK Snap) | 81.25 | 0.85 | 32 | 2.83 | 1 |
| Region Competition Snakes (ITK Snap) | 79.85 | 1.27 | 20 | 1.27 | 4 |
| Edge Based Snake (ITK Snap) | 80.79 | 1.80 | 19 | 2.00 | 4 |
| Manual Paint and Contouring (Seg3D) | 80.26 | 1.29 | 31 | 2.35 | 2 |
| Speedline (Seg3D) | 79.67 | 1.36 | 20 | 1.87 | 3 |
| CAC Toolkit (our method) | 80.02 | 0.68 | 5 | 0.35 | 1 |
| Average | 80.31 | 1.21 | 21.2 | 1.78 |  |

*SD: standard deviation
